# Predictors of social inclusion among adults with severe mental illness: Results of a cross-sectional study

**DOI:** 10.1177/00207640251350218

**Published:** 2025-07-31

**Authors:** Mara Ohlhoff, Alexander Pabst, Johanna Breilmann, Thomas Becker, Andreas Allgöwer, Reinhold Kilian, Alkomiet Hasan, Peter Falkai, Klemens Ajayi, Theresa Halms, Peter Brieger, Karel Frasch, Stephan Heres, Markus Jäger, Andreas Küthmann, Albert Putzhammer, Steffi G. Riedel-Heller, Bertram Schneeweiß, Michael Schwarz, Markus Kösters, Uta Gühne

**Affiliations:** 1 Institute of Social Medicine, Occupational Health and Public Health (ISAP), Medical Faculty, University of Leipzig, Germany; 2 Department of Psychiatry and Psychotherapy II, Ulm University, Günzburg, Germany; 3 Department of Psychiatry and Psychotherapy, Medical Faculty, University of Leipzig, Germany; 4 Institute for Epidemiology and Medical Biometry, Ulm University, Germany; 5 Department of Psychiatry, Psychotherapy and Psychosomatics, Medical Faculty, University of Augsburg, Germany; 6 German Centre for Mental Health, Munich-Augsburg, Augsburg, Germany; 7 Department of Psychiatry and Psychotherapy, University Hospital Munich, Germany; 8 Kbo-Isar-Amper Hospital, Region Munich, Germany; 9 Department of Psychiatry, Psychotherapy and Psychosomatics, District hospital Donauwörth, Germany; 10 Department of Psychiatry, Psychotherapy and Psychosomatics, District hospital Kempten, Germany; 11 Department of Psychiatry, Psychotherapy and Psychosomatics, District hospital Memmingen, Germany; 12 Department of Psychiatry, Psychotherapy and Psychosomatics, District hospital Kaufbeuren, Germany; 13 Center for Evidence-Based Healthcare, University Hospital Dresden and Medical Faculty Carl Gustav Carus, Dresden University of Technology, Germany

**Keywords:** Social inclusion, severe mental illness, employment status, intimate relationships, living situation

## Abstract

**Background:**

Promoting social inclusion is crucial for people living with severe mental illness (SMI), who often experience high levels of social exclusion. However, research that uses a psychometric social inclusion measure to identify factors that determine varying levels of social inclusion in individuals with SMI is scarce.

**Aims:**

This study aimed to examine to what extent people with SMI feel socially included and to identify factors associated with perceived social inclusion among people with SMI.

**Method:**

A cross-sectional multicenter investigation of psychiatric inpatients and day hospital patients with SMI aged 18 to 65 years (*n* = 358) was conducted. Perceived social inclusion, sociodemographic, and clinical characteristics were assessed using the Measure of Participation and Social Inclusion for Use in People with a Chronic Mental Disorder (F-INK). Hierarchical multiple linear regression was performed to analyse the association between social inclusion and potential predictors.

**Results:**

The participants’ overall level of social inclusion was moderate (F-INK social inclusion total score *M* = 1.9, *SD* = 0.6). Age, relationship status, diagnostic group, employment status, and living situation emerged as predictors of social inclusion. Greater subjective social inclusion was predicted by older age (*p* = .027), being in a committed intimate relationship (*p* = .037), diagnosis of schizophrenia spectrum disorder (compared to diagnosis of depression, *p* = .020), being competitively employed or in education (compared to being in sheltered employment, *p* = .022; compared to being unemployed or receiving a disability pension, *p* = .007), and living with other people (*p* = .042).

**Conclusions:**

The results confirm deficiencies in social inclusion of people with SMI. Individuals with SMI who are younger, single, have a diagnosis of depression, are in sheltered employment, are unemployed or receiving a disability pension, and are living alone seem to be particularly at risk of experiencing low social inclusion. These findings highlight the importance of psychosocial interventions in rehabilitative mental healthcare.

## Trial registration:

The study was registered in the German Clinical Trials Register (DRKS) and the WHO International Clinical Trials Registry Platform (ICTRP) under registration number DRKS00015801 (https://drks.de/search/en/trial/DRKS00015801; https://trialsearch.who.int/?TrialID=DRKS00015801) before start of recruitment (date of registration: 21/02/2019).

## Background

The right of every person to social inclusion irrespective of physical or mental impairments is enshrined in the United Nations Convention on the Rights of Persons with Disabilities (UN CRPD; 2006), and its implementation has been committed to by nations worldwide. Nevertheless, people with mental illness, especially people with severe mental illness (SMI), experience high levels of exclusion. Relative to the general population, they participate in social leisure activities less often (Boardman, 2011; Richter & Hoffmann, 2019), report feeling lonelier (Meltzer et al., 2013; Richter & Hoffmann, 2019), are less likely to be married and more likely to be divorced (Breslau et al., 2011; Richter et al., 2006; Richter & Hoffmann, 2019). This holds true for various further domains, including lower income levels (Jenkins et al., 2008; Luciano & Meara, 2014; Richter et al., 2006; Richter & Hoffmann, 2019), higher rates of people being in debt (Jenkins et al., 2008), higher rates of people living in unstable accommodation (Gardner et al., 2019; Social Exclusion Unit, 2004), and higher unemployment rates (Luciano & Meara, 2014; Richter et al., 2006; Richter & Hoffmann, 2019).

Investigating social inclusion is hampered by a lack of conceptual clarity. Despite a growing body of literature on social inclusion, a consensus definition does not exist (K. M. Filia et al., 2018; O’Donnell et al., 2018). Hence, research aiming to investigate social inclusion in mental health contexts has included a wide variety of operationalisations (Baumgartner & Burns, 2014). In many cases researchers have drawn upon proxies, such as a numerical count of activities (De Heer-Wunderink et al., 2012), employment status (Revier et al., 2015), or measures of related concepts (e.g. social functioning; Adamus et al., 2022). Theoretically underpinned and psychometrically sound instruments explicitly designed to measure social inclusion have long been missing. Over the past decade, several research teams have begun to address this gap. Nevertheless, there is no gold standard measure of social inclusion (Cordier et al., 2017). Due to the absence of a universal definition, social inclusion measures vary widely in terms of the thematic areas they cover (e.g. attendance of community spaces and social activities, financial and material resources, active citizenship, connectedness) and the relative emphasis they place on objective versus subjective criteria (objective: e.g. activities engaged in, access to material goods; subjective: e.g. satisfaction with opportunities, experience of feeling accepted, reasons for non-participation) (Coombs et al., 2016; K. Filia et al., 2022; Huxley et al., 2012; Mezey et al., 2013, 2020; Schützwohl et al., 2017; Secker et al., 2009; Wilson & Secker, 2015; among these, only one instrument [Schützwohl et al., 2017] is available in German).

Studies that use a psychometric social inclusion measure for the purpose of identifying determinants of social inclusion in individuals with SMI are scarce. Some factors have been shown to relate to greater or lower social inclusion (Mezey et al., 2022; Schützwohl, 2017), or to the change in a person’s level of social inclusion after onset of psychosis (Killaspy et al., 2014). However, limited evidence concerning each of these factors and differing conceptualisations of social inclusion impede the synthesis of results. To draw well-founded conclusions, further research is required.

Generating further information on the factors determining varying levels of social inclusion among people with SMI is needed in order to identify particularly vulnerable subgroups, as well as to expand the empirical foundation for developing and implementing interventions in rehabilitative mental health care. Therefore, this study aimed to answer the following questions: (1) To what extent do adult people with SMI feel socially included? (2) What factors are associated with social inclusion among these individuals with SMI?

## Methods

### Design and Setting

This study was based on data from an observational, cross-sectional investigation on patients with SMI, conducted in the context of a larger, multicenter study project (Implementation Status of the German Guideline for Psychosocial Interventions for Patients with Severe Mental Illness [IMPPETUS]; Breilmann et al., 2018). The project was approved by the Ulm University ethics committee (no. 463/18). Recruitment and data collection took place between March and September 2019 in 10 departments of psychiatry and psychotherapy providing in- and outpatient psychiatric care for people with mental illness in the acute phases of their illness. The catchment areas included metropolitan (Augsburg, Munich), middle-urban (Kempten, Memmingen) and rural (Donauwoerth, Guenzburg, Kaufbeuren, Taufkirchen) regions in Bavaria, Southern-Germany. Eligible inpatients and day hospital patients were invited to participate in the study and screened by trained study personnel shortly after admission. Comprehensive data collection interviews were conducted by trained study personnel and took place during the patient’s inpatient or day hospital stay shortly before discharge. All participants provided written informed consent.

The present study was reported in accordance with the STROBE statement (von Elm et al., 2008).

### Inclusion Criteria

To identify patients with SMI, the following criteria were used:

(I) diagnosis of schizophrenia, schizotypal or delusional disorder (ICD-10 F2x), bipolar disorder (ICD-10 F30, F31), or depressive disorder (ICD-10 F32, F33),
(II) duration of psychiatric illness ⩾2 years, and
(III) substantial impact on activities of daily life and social functioning, defined by Global Assessment of Functioning (GAF; Jones et al., 1995) and Health of the Nation Outcome Scales (Wing et al., 1998; German version: HoNOS-D, Andreas et al., 2010) scores.

These criteria are based on the definition of SMI used by the German guidelines for psychosocial interventions in severe mental illness (Gühne et al., 2019), which is in line with the European tradition of defining SMI (Delespaul, 2013; Gühne et al., 2015; Parabiaghi et al., 2006; Ruggeri et al., 2000; Schinnar et al., 1990). In contrast to the guidelines’ definition, inclusion criteria were limited to three diagnostic groups, in order to obtain a more homogenous sample.

(III) was operationalised as a GAF score ⩽60 and the HoNOS-D scores fulfilling one of two conditions, (a) a score of ⩾2 on one of the items of the subscale for symptomatic problems (items 6–8) and a score of ⩾2 on each of the items of the subscale for social problems (items 9–12) or (b) a score of ⩾3 on at least one of these items (items 9–12).

The GAF reflects a person’s level of social, psychological, and occupational functioning, as a score between 1 (very severely impaired functioning) and 100 (excellent level of functioning), further taking into account psychiatric symptom severity. A GAF score ⩽60 indicates moderately to very severely impaired social functioning (cut-off based on Ruggeri et al., 2000).

The HoNOS-D is a 12-item instrument for assessing behaviour, impairment, symptoms, and social functioning of individuals with mental illness, rating different areas on a scale from 0 (no problem in this field) to 4 (severe to very severe problems in this field). Scores ⩾2 represent clinically significant problems (Andreas, 2005; Burgess et al., 2009).

Duration of illness was taken from medical records or from information provided by the treating physician. The diagnosis was established by the treating psychiatrist at the outset of the inpatient or day hospital treatment.

In addition to the SMI criteria (I–III), the following inclusion criteria were applied:

(IV) aged 18 to 65 years,
(V) capacity to give informed consent, and
(VI) German language proficiency sufficient to understand questionnaires and questions asked.

### Measures

#### Social Inclusion

To measure social inclusion, the module on social inclusion from the Measure of Participation and Social Inclusion for Use in People with a Chronic Mental Disorder (F-INK; Schützwohl et al., 2017) was used. The F-INK is a modular questionnaire, designed to measure participation and social inclusion in individuals with a chronic mental disorder. It consists of nine modules covering the nine key variables of the theoretical model of social inclusion by Schützwohl et al. (2017). Schützwohl et al. posit that sociodemographic and clinical variables serve as the foundation upon which facilitating factors and resulting variables interact in a reinforcing manner, with a variety of interconnections. The modules can be used independently from each other.

The concept of social inclusion, as operationalised by the F-INK social inclusion module, is defined as a subjective perception of involvement and belonging. The total score for social inclusion is calculated as the mean of ratings of seven universally applicable items of the module. These items are rated on a 4-point Likert scale ranging from 0 (not at all) to 3 (a lot). They comprise statements regarding the extent to which respondents feel respected and accepted in various life domains (e.g. neighbourhood, family, society; items 1–5, 12) and one statement about the extent of feeling like belonging to society in general (item 13). (Social inclusion module items 6–11 are not included in the total score as they only apply to certain subgroups of people, e.g. individuals living in supported housing facilities.) The social inclusion total score has been shown to have substantial internal consistency (Cronbach’s α = .77; Schützwohl et al., 2017).

#### Sociodemographic and Clinical Data

Sociodemographic data (age, gender, migration background, relationship status, employment status, type of housing, living situation) and clinical data (age at onset of mental problems, presence of a chronic physical illness) were gathered using items retrieved from the modules on sociodemographic data, clinical data, occupation, and living situation of the F-INK (Schützwohl et al., 2017). Participants were considered to have a migration background if they had immigrated themselves or if at least one of their parents had immigrated. Information on employment status was classified into four categories as follows: (a) being competitively employed or in education, (b) being in sheltered employment, (c) looking after the home and caring for the family, being on parental leave, doing unpaid voluntary work, or being retired for reasons of age, and being non-assignable to one of the two aforementioned categories, (d) receiving a disability pension or being unemployed, and being non-assignable to one of the three aforementioned categories. Housing types were collapsed into the following four categories: living independently, being the recipient of a supported independent living programme, living in a supported housing facility, or being unhoused. A further item on living situation distinguished whether a person was living alone or with other people (e.g. family, housemates).

Clinical data was complemented by GAF score and diagnostic group; both were assessed during screening procedures.

### Statistical Analysis

Descriptive statistics were calculated in the form of absolute frequencies and percentages for nominal categorical data, means and standard deviations for normally distributed continuous data, and medians and interquartile ranges for ordinal categorical or not normally distributed continuous data. Continuous variables were checked for normal distribution graphically.

To analyse the association between social inclusion and potential determinants a three-step hierarchical multiple linear regression was used. In a first step, Model 1 examined the relation between social inclusion and sociodemographic variables, that is, age, gender, migration background, and status of being in a committed intimate relationship. Model 2 assessed the additional impact of clinical variables. In Model 3, employment status, type of housing, and living situation were entered into the model, representing three of the so-called facilitating factors identified by Schützwohl et al. (2017). The stepwise approach permitted the examination of the contribution, or additional contribution, of sociodemographic, clinical, and facilitating factors separately. Unstandardised regression coefficients (*B*) with 95% confidence intervals (CI), and standardised regression coefficients (β) were computed. Diagnostic group, employment status, and type of housing were entered into the model as dummy variables, with schizophrenia spectrum disorder (F2x), competitive employment / education, and living independently, respectively, forming the reference categories. To test their overall significance as predictors Wald χ² tests were performed. For all included variables it was verified that there were no multicollinearity concerns (variance inflation factor, VIF < 5; Marcoulides & Raykov, 2019). *F* tests were conducted for each model to ensure the models’ significance. Adjusted *R*^2^ values were calculated to assess proportions of explained variance.

Cases of missing data were handled by case-wise deletion. Two-sample independent *t* tests and Pearson’s chi-squared test were performed to test for differences in age, gender, and GAF, between cases included in the regression analysis and removed cases. The same procedures were applied to test for systematic biases resulting from excluding individuals who were missing essential data on social inclusion.

A significance level of α = .05 was used for all statistical tests. All statistical analyses were conducted using IBM SPSS Statistics for Windows, Version 29.

## Results

### Participants

**Figure 1.**
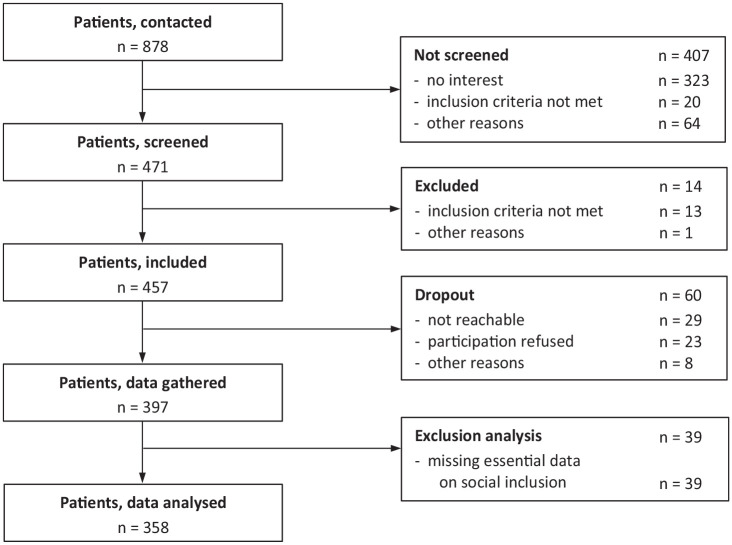
Flow chart of participants.

Data were gathered for 397 participants who met the inclusion criteria, and 39 participants were excluded from the analyses due to missing essential data on social inclusion (more than one item from the social inclusion scale and/or the general social inclusion item [item 13] was missing). Thus, the analyses were conducted with data of 358 participants (Figure 1).

### Participants Characteristics

Participants were aged between 18 and 65 years, with a mean age of 42.5 years (*SD* 13.1 years). Of 358 study participants included in the analyses in total, 201 (56.1%) were female. 149 participants (42.9%) reported being in a committed intimate relationship. The majority of patients had a diagnosis of depressive disorder (*n* = 213, 59.5%), followed by the group of patients diagnosed with a schizophrenia spectrum disorder (*n* = 109, 30.4%). A mean GAF score of 42.9 (*SD* 9.6, range 19–60) indicated severe impairment of functioning. 135 people (41.5%) were competitively employed or enrolled in education, while 146 (44.9%) were receiving a disability pension or unemployed. Most participants (*n* = 307, 86.7%) were living independently without support. More than half of the participants (*n* = 206, 58.4%) reported living with other people (Table 1).

**Table 1.** Sociodemographic and clinical characteristics of study participants (*n* = 358).

Characteristic	All patients (*n* = 358)
Age (years), mean (*SD*) (*n* = 357)^a^	42.5 (13.1)
Gender, *n* (%)	
Female	201 (56.1)
Male	157 (43.9)
Migration background, *n* (%) (*n* = 356)	
No	295 (82.9)
Yes	61 (17.1)
Committed intimate relationship, *n* (%) (*n* = 347)	
No	198 (57.1)
Yes	149 (42.9)
Age at onset of mental problems (years), median (*IQR*) (*n* = 336)	24 (17–37)
Diagnostic group, *n* (%)	
Schizophrenia spectrum disorder^b^	109 (30.4)
Depressive disorder^c^	213 (59.5)
Bipolar disorder^d^	36 (10.1)
Comorbid chronic physical illness, *n* (%) (*n* = 357)	
No	183 (51.3)
Yes	174 (48.7)
GAF, mean (*SD*) (*n* = 351)^a^	42.9 (9.6)
Employment status, *n* (%) (*n* = 325)	
Competitive employment / in education^e^	135 (41.5)
Sheltered employment	15 (4.6)
Caring for the house and the family / parental leave / voluntary work / retired	29 (8.9)
Disability pension / unemployed	146 (44.9)
Type of housing, *n* (%) (*n* = 354)	
Independent living	307 (86.7)
Supported independent living	11 (3.1)
Supported housing facility	25 (7.1)
Unhoused	11 (3.1)
Living situation, *n* (%) (*n* = 353)	
Alone	147 (41.6)
With others	206 (58.4)

*Note*. Varying subsample sizes due to missing information are displayed in parentheses. GAF = Global Assessment of Functioning.

a Fulfilment of inclusion criteria was documented for all participants; however, in eight cases specific values are missing for age (*n* = 1) or GAF (*n* = 7).

b ICD-10 F2x.

c ICD-10 F32, F33.

d ICD-10 F30, F31.

e Self-employed individuals and individuals employed in the open labour market were considered to be competitively employed, including full-time, part-time, and marginal part-time work; being in education included being in school, vocational training, or university.

There was no significant difference in age, *t*(393) = 1.01, *p* = .314, or gender, χ²(1) = 0.91, *p* = .341, between individuals excluded from the analyses due to missing essential data on social inclusion (*n* = 39; Figure 1) and those included in the analyses. Whereas, individuals who were missing essential data on social inclusion scored on average lower on the GAF (*M* 37.4, *SD* 10) compared to those included in the analyses, *t*(388) = −3.38, *p* < .001.

### Social Inclusion

Social inclusion total scores ranged from 0 to 3, with a mean of 1.9 (*SD* 0.6). Normal distribution of total scores indicated appropriateness of linear modelling. Further descriptive statistics on social inclusion are reported in Table 2.

**Table 2.** Social inclusion of study participants (*n* = 358).

F-INK social inclusion module universally applicable items	*Mdn* (*IQR*)	*M* (*SD*)
To what extent do you feel respected and accepted in your living environment, i.e. by your neighbours? (*n* = 344)	2 (1–3)	
To what extent do you feel respected and accepted by your closest family? (*n* = 357)	2 (1–3)	
To what extent do you feel respected and accepted by your extended family? (*n* = 344)	2 (1–3)	
To what extent do you feel respected and accepted during your activities of daily self-care (e.g. while doing the shopping)? (*n* = 355)	2 (2–3)	
To what extent do you feel respected and accepted during your leisure time activities? (*n* = 348)	2 (2–3)	
To what extent do you feel respected and accepted by society in general? (*n* = 355)	2 (1–2)	
To what extent do you feel like belonging to society in general?	1 (1–2)	
**F-INK social inclusion total score**		1.9 (0.6)

*Note*. Varying subsample sizes due to missing information are displayed in parentheses. F-INK = Measure of Participation and Social Inclusion for Use in People with a Chronic Mental Disorder.

### Predictors of Social Inclusion

Case-wise deletion resulted in 21.8% of cases being removed from the regression analysis. Testing for group differences revealed no systematic biases due to the removal of cases with missing data.

In Model 1, being in a committed intimate relationship, as compared to being single, predicted greater social inclusion (*p* < .001). This relationship remained the same when clinical variables were added to the model. Further, in Model 2 a higher GAF score was related to greater social inclusion (*p* = .004), adjusted for sociodemographic characteristics. Of the facilitating variables entered into Model 3, employment status (*p* = .010) and living situation were significant predictors of a person’s level of social inclusion, adjusted for sociodemographic and clinical characteristics. Being in sheltered employment (*p* = .022) and being unemployed or receiving a disability pension (*p* = .007) were related to lower social inclusion, compared to being competitively employed or in education. Further, living with other people, compared to living alone, was associated to greater social inclusion (*p* = .042). The status of being in a committed intimate relationship, as opposed to being single, remained significant as a predictor in Model 3 (*p* = .037). Furthermore, Model 3 indicated an association of older age to greater social inclusion (*p* = .027). With regard to the clinical variables, diagnostic group was a significant predictor in Model 3 (*p* = .021), with a diagnosis of depression predicting lower social inclusion compared to a schizophrenia spectrum diagnosis (*p* = .020). The GAF score lost its significance as a predictor in Model 3, when facilitating factors were accounted for (Table 3).

**Table 3.** Hierarchical multiple linear regression: Predicting social inclusion (F-INK social inclusion total score) from sociodemographic, clinical, and facilitating factors (*n* = 280).

Predictor variable	Model 1(*F* = 5.42***, adjusted *R*^2^ = 0.060)				Model 2 (*F* = 4.00***, adjusted *R*^2^ = 0.088)			Model 3 (*F* = 3.56***, adjusted *R*^2^ = 0.128)		
	*B*		95% CI for *B*	β	*B*	95% CI for *B*	β	*B*	95% CI for *B*	β
Age	0.01		[0.00, 0.01]	.11	0.01	[0.00, 0.01]	.13	0.01*	[0.00, 0.02]	.18
Gender										
Female	Ref.									
Male	0.05		[−0.10, 0.19]	.04	0.02	[−0.13, 0.16]	.02	−0.01	[−0.15, 0.14]	−.01
Migration background										
No	Ref.									
Yes	0.10		[−0.09, 0.29]	.06	0.14	[−0.06, 0.33]	.08	0.13	[−0.07, 0.32]	.08
Committed intimate relationship										
No	Ref.									
Yes	0.28***		[0.13, 0.43]	.22	0.28***	[0.13, 0.43]	.22	0.17*	[0.01, 0.34]	.14
Age at onset of mental problems					0.00	[−0.01, 0.01]	−.05	0.00	[−0.01, 0.00]	−.08
Diagnostic group					χ² = 4.71			χ² = 7.72*		
Schizophrenia spectrum disorder^a^					Ref.					
Depressive disorder^b^					−0.12	[−0.28, 0.04]	−.10	−0.20*	[−0.36, −0.03]	−.16
Bipolar disorder^c^					0.10	[−0.15, 0.36]	.05	0.02	[−0.25, 0.28]	.01
Chronic physical illness										
No					Ref.					
Yes					−0.04	[−0.18, 0.11]	−.03	0.01	[−0.14, 0.16]	.01
GAF					0.01**	[0.00, 0.02]	.18	0.01	[0.00, 0.02]	.12
Employment status								χ² = 11.28*		
Competitive employ-ment / in education								Ref.		
Sheltered employment								−0.42*	[−0.78, −0.06]	−.14
Unpaid work / parental leave/retired								−0.25	[−0.54, 0.04]	−.12
Disability pension / unemployed								−0.22**	[−0.39, −0.06]	−.18
Type of housing								χ² = 4.32		
Independent living								Ref.		
Supported independent living								−0.08	[−0.53, 0.38]	−.02
Supported housing facility								−0.24	[−0.51, 0.04]	−.10
Unhoused								−0.31	[−0.79, 0.18]	−.07
Living situation										
Alone								Ref.		
With others								0.17*	[0.01, 0.33]	.14

*Note*. F-INK = Measure of Participation and Social Inclusion for Use in People with a Chronic Mental Disorder. GAF = Global Assessment of Functioning. CI = confidence interval. Ref. = reference category. χ² represents Wald tests’ results. Bold type indicates statistical significance: *p < .05, **p < .01, ***p < .001.

a ICD-10 F2x.

b ICD-10 F32, F33.

c ICD-10 F30, F31.

## Discussion

### Social Inclusion

The results confirm deficiencies in social inclusion of people with SMI. The average level of social inclusion in this sample of adult psychiatric inpatients and day hospital patients with SMI was moderate. Our finding is similar to the mean F-INK social inclusion total scores reported for individuals with SMI in previous studies (Mergel & Schützwohl, 2021; Schützwohl, 2017; Wartmann et al., 2019). In accordance with international evidence that indicates lower social inclusion of people with SMI compared to the general population (K. Filia et al., 2022; Huxley et al., 2012; Mezey et al., 2020), the mean F-INK social inclusion total score found in the present study’s SMI sample was lower than values identified in healthy samples (Mergel & Schützwohl, 2021; Schützwohl et al., 2017). Concerning the individual items, statements regarding society in general were rated lower than those regarding more specific life domains. The same tendency was found in previous research (Schützwohl, 2017).

### Predictors of Social Inclusion

In keeping with previous evidence (Schützwohl, 2017), being in a committed intimate relationship was related to a sense of greater social inclusion. Singles with SMI reported feeling less socially included, which is important, considering that a large proportion of individuals with SMI are single (White et al., 2021). Despite the domain of intimate relationships being one of the areas of unmet needs most reported by people with SMI (Eager et al., 2023; McCann, 2010; Werner, 2012), there is a great lack of interventions directly focussed on addressing this need (Caiada et al., 2024; Cloutier et al., 2021). The present study’s finding emphasises the relevance of developing such interventions for people with SMI that aim to support individuals in engaging in and maintaining stable intimate relationships.

Further, employment status predicted social inclusion. Being unemployed or receiving a disability pension, and enrolment in sheltered employment were associated with lower social inclusion. In contrast, individuals who were caring for the house and the family, who were on parental leave, doing unpaid voluntary work, or who were retired for reasons of age, did not significantly report less social inclusion than individuals engaged in competitive employment or enrolled in education. This is in line with findings in a first episode psychosis cohort, where at follow-up greater social inclusion correlated with being currently employed and with duration of paid employment or non-paid work since first episode psychosis (Turner et al., 2015). Our results highlight the importance of vocational rehabilitation interventions, given the high rates of people with SMI who are not competitively employed (Dehn et al., 2024; Gühne et al., 2022; Marwaha et al., 2007) and especially when considering the strong desire to work among patients with SMI (Gühne et al., 2021). The finding that people in sheltered employment felt less socially included than those in competitive employment gives additional weight to prioritising supported employment interventions over traditional approaches (including sheltered employment) in rehabilitative mental health care for people with SMI. There is strong international evidence for beneficial effects of supported employment compared to traditional ‘first train, then place’ strategies, with regard to various outcomes, such as obtainment and maintenance of competitive employment or reduction of psychiatric hospital admissions (Hoffmann et al., 2014; Modini et al., 2016; Suijkerbuijk et al., 2017).

Moreover, living with others predicted greater social inclusion. The association of living alone and lower subjective social inclusion should be considered in the context of support needs of people with SMI who are living independently. Housing type itself, however, did not impact social inclusion significantly among our sample. Independent living, as compared to institutionalised supported accommodation, is related to greater autonomy and participation in social activities, and at the same time, it involves increased perceived loneliness, lower satisfaction with daily life, and harder-to-access social care, healthcare, and other interventions (De Heer-Wunderink et al., 2012; Harvey et al., 2012; Killaspy et al., 2016; Steinhart, 2018). A systematic review on outcomes of supported independent housing and institutionalised accommodation settings suggests no clear trends in favour of one or the other housing type for not-unhoused people with SMI (Richter & Hoffmann, 2017a). In the light of a strong preference for independent living (Lamp et al., 2025; Richter & Hoffmann, 2017b), strengthening the care providers’ supporting structures to enable adequate support that, in quantity and quality, matches the individual needs of people living independently, is crucial for genuinely providing equal opportunities for people with SMI to choose a place of residence and with whom to live, as stated in the UN CRPD (2006).

The relevance of the facilitating factors (in accordance with the model of social inclusion by Schützwohl et al., 2017) was further emphasised by the finding that a seemingly significant impact of the level of psychosocial functioning, operationalised by GAF score, turned out to be insignificant when employment status, housing type, and living situation were entered to the regression model. Therefore, effective vocational rehabilitation and suiting interventions related to living situation may significantly improve social inclusion, even in cases of low levels of functioning.

Lastly, in the final model, older age and diagnostic group predicted social inclusion. The association between older age and greater social inclusion contrasts with previous evidence, demonstrating younger age to be related to greater social inclusion (Mezey et al., 2022). A reason for these seemingly contradictory results might be the different conceptualisation of social inclusion of the instruments used for measurement. The Social Inclusion Questionnaire User Experience (SInQUE; Mezey et al., 2013), used by Mezey et al. (2022), mainly captures objective aspects (e.g. activities a person participates in; accounting for subjective aspects in the form of a ‘not interested in’ response option). Older people might objectively participate less, but at the same time subjectively experience more feelings of belonging, as compared to younger people. Similarly, conceptual differences might be one reason for the differing of our results from those of Mezey et al. (2022) concerning the impact of diagnostic group. The authors did not find an association between diagnostic group and total level of social inclusion. Whereas, in our investigation, when social inclusion was measured as a self-rated subjective feeling, a negative depressive mindset could have led to the reporting of lower social inclusion by patients with a diagnosis of depression, as compared to patients diagnosed with a schizophrenia spectrum disorder. In addition, a reason for those differing results could be different categorising of diagnostic groups (depression, anxiety, and obsessive-compulsive disorders were collapsed into one category for the analyses by Mezey et al.).

Low proportions of explained variance in the regression models indicated the contribution of further factors to explaining varying levels of social inclusion.

### Limitations and Strengths

Certain limitations must be considered when interpreting the results of this study. First, there are concerns regarding generalisability that need to be addressed. The most severely impaired individuals could have been excluded from this investigation. Individuals excluded from the analyses due to missing essential data on social inclusion scored lower on the GAF than those included in the analyses, which suggests possible barriers in the data collection process, leading to biases concerning incomplete data. Recruitment and inclusion criteria might also have led to the exclusion of the most severely impaired (e.g. ability to give informed consent). Further, recruitment was limited to adult psychiatric inpatients and day hospital patients with SMI in Bavaria, Southern-Germany. Perspectives on mental illness vary across cultures (Altweck et al., 2015; Fekih-Romdhane et al., 2023; Lauber & Rössler, 2007), as do perceptions of social inclusion (Chan et al., 2014; Chiu et al., 2016). The generalisability of our results to other cultural contexts, as well as to settings other than the acute inpatient or day hospital treatment, remains unexamined. Second, this investigation could be subject to limitations concerning objectivity, as most data collection was based on self-reported information. Third, the data should only be interpreted in a correlative way. Further research is needed to understand direction of causality and underlying mechanisms. Fourth, particular attention should be given to the measurement of social inclusion. The F-INK social inclusion module captures social inclusion as subjective experience of feeling respected, accepted, and like belonging to society. Other, more objective aspects (e.g. participation in activities, employment) are seen as separate factors by the F-INK’s authors, and hence, are captured by separate modules. Social inclusion measures vary widely in terms of thematic focus, thematic areas included, and proportion of objective and subjective aspects included (Coombs et al., 2016; K. Filia et al., 2022; Huxley et al., 2012; Mezey et al., 2013; Secker et al., 2009). As discussed above, differing conceptualisations of social inclusion make it hard to compare and synthesise results from studies using different measures, limiting general conclusions. However, given the relevance of this topic for those who are affected, it is not an acceptable option to postpone research on social inclusion of people with SMI until a consensus definition and gold standard measure of social inclusion are adopted. To our knowledge, the F-INK is the only German language instrument that is theoretically underpinned and explicitly designed to measure social inclusion. It has been subject to preliminary testing of psychometric properties (Schützwohl et al., 2017), further testing on a larger sample is ongoing (University Hospital Dresden Carl Gustav Carus, n.d.). The present study is one out of very few studies that aim to identify factors associated with social inclusion in individuals with SMI and that use a theoretically and psychometrically sound social inclusion measure for that purpose.

## Conclusion

Social inclusion of people with SMI needs to be improved. According to our findings, individuals who are single, who are unemployed or receiving a disability pension, who are in sheltered employment, who are living alone, who are younger, and individuals with a diagnosis of depression, appear to be particularly at risk to experience low social inclusion. It is important to account for these subgroups’ particular vulnerability, and to consider these findings in development and implementation of psychosocial interventions. Further clarifying of the factors that determine varying levels of social inclusion among people with SMI, causality, and underlying mechanisms should be subject to future research.
